# Low-Lying Electronic States of the Nickel Dimer

**DOI:** 10.3389/fchem.2021.678930

**Published:** 2021-05-13

**Authors:** Patrick K. Tamukong, Mark R. Hoffmann

**Affiliations:** Chemistry Department, University of North Dakota, Grand Forks, ND, United States

**Keywords:** nickel dimer, metal dimers, multireference perturbation theory, generalized Van Vleck perturbation theory, electronic structure calculations

## Abstract

The generalized Van Vleck second order multireference perturbation theory (GVVPT2) method was used to investigate the low-lying electronic states of Ni_2_. Because the nickel atom has an excitation energy of only 0.025 eV to its first excited state (the least in the first row of transition elements), Ni_2_ has a particularly large number of low-lying states. Full potential energy curves (PECs) of more than a dozen low-lying electronic states of Ni_2_, resulting from the atomic combinations ^3^F_4_ + ^3^F_4_ and ^3^D_3_ + ^3^D_3_, were computed. In agreement with previous theoretical studies, we found the lowest lying states of Ni_2_ to correlate with the ^3^D_3_ + ^3^D_3_ dissociation limit, and the holes in the d-subshells were in the subspace of delta orbitals (i.e., the so-dubbed δδ-states). In particular, the ground state was determined as X ^1^Γ_g_ and had spectroscopic constants: bond length (*R*_e_) = 2.26 Å, harmonic frequency (ω_e_) = 276.0 cm^−1^, and binding energy (*D*_e_) = 1.75 eV; whereas the 1 ^1^Σ_g_^+^ excited state (with spectroscopic constants: *R*_e_ = 2.26 Å, ω_e_ = 276.8 cm^−1^, and *D*_e_ = 1.75) of the ^3^D_3_ + ^3^D_3_ dissociation channel lay at only 16.4 cm^−1^ (0.002 eV) above the ground state at the equilibrium geometry. Inclusion of scalar relativistic effects through the spin-free exact two component (sf-X2C) method reduced the bond lengths of both of these two states to 2.20 Å, and increased their binding energies to 1.95 eV and harmonic frequencies to 296.0 cm^−1^ for X ^1^Γ_g_ and 297.0 cm^−1^ for 1 ^1^Σ_g_^+^. These values are in good agreement with experimental values of *R*_e_ = 2.1545 ± 0.0004 Å, ω_e_ = 280 ± 20 cm^−1^, and *D*_*0*_ = 2.042 ± 0.002 eV for the ground state. All states considered within the ^3^F_4_ + ^3^F_4_ dissociation channel proved to be energetically high-lying and van der Waals-like in nature. In contrast to most previous theoretical studies of Ni_2_, full PECs of all considered electronic states of the molecule were produced.

## Introduction

Since Ni_2_ has few holes in otherwise complete subshells, one might expect theoretical studies of Ni_2_ to be less complicated than for other first row transition metal dimers, like Cr_2_ where one has many more possibilities of distributing 12 electrons in 12 valence orbitals. Reported information on Ni_2_, however, proves the contrary. For example, the exact symmetry of the *ground* electronic state of Ni_2_ is still debated: Different studies have reported different space and spin symmetries for the molecule’s ground term.

Experimental data on Ni_2_ is sparse and the true ground state of the molecule is not unequivocally accepted. From the analysis of electronic absorption bands of Ni_2_ in the visible spectral region in argon matrices, De Vore et al. (De Vore et al., 1975) determined ω_e_ = 192 cm^−1^, whereas a frequency of 380.9 cm^−1^ was found in solid argon matrix (Ahmed and Nixon, 1979). The latter result was later criticized by Rasanen et al. (Rasanen et al., 1987) In photoelectron spectroscopic studies of Ni_2_^-^, ω_e_ = 280 ± 20 cm^−1^ was determined for the lowest electronic state of Ni_2_ (Ho et al., 1993). Second and third law analyses of information derived from a combination of Knudsen effusion and mass‐spectrometric techniques led to a binding energy of *D*_*0*_ = 2.03 ± 0.30 eV (second law result) and *D*_*0*_ = 2.36 ± 0.22 eV (third law result) for ground state Ni_2_ (Kant, 1964). By using time-delayed resonant two-photon ionization, Morse et al. (Morse et al., 1984) determined *D*_*0*_ = 2.068 ± 0.010 eV and *R*_e_ = 2.200 ± 0.007 Å for the lowest state of Ni_2_, but assigned as either ^3^Γ_u_ or ^1^Γ_g_. Also from two-photon ionization studies on supersonic jet-cooled Ni_2_ in argon carrier gas, Pinegar et al. (Pinegar et al., 1995) determined *D*_*0*_ = 2.042 ± 0.002 eV and *R*_e_ = 2.1545 ± 0.0004 Å for the lowest state of Ni_2_ but were unable to ascertain the symmetry of this state.

Theoretical studies of the electronic states of Ni_2_ are complicated by the fact that the first excited state of the Ni atom, ^3^D_3_ (3d^9^4s^1^), is only 0.025 eV above the ^3^F_4_ (3d^8^4s^2^) ground atomic state, which is the least excitation energy of any of the first row of transition elements (Harrison, 2000; Kramida et al., 2013). This low promotion energy supports the existence of several low-lying molecular states of the Ni_2_ molecule resulting from the ^3^F_4_ + ^3^F_4_ and ^3^D_3_ + ^3^D_3_ atomic combinations. For example, limited configuration interaction (CI) calculations (Shim et al., 1979) found 84 states of Ni_2_, corresponding to the ^3^F_4_ + ^3^F_4_ dissociation limit, to lie within an energy range of only 300 K (0.026 eV), and 45 states, also within a narrow energy range, to correlate with the ^3^D_3_ + ^3^D_3_ dissociation asymptote. Melius et al. (Melius et al., 1976) also noted that the manifold of electronic states within 0.50 eV of the ground state of Ni_2_ was dense and complex.

Since the fully filled 4s-subshell of the ^3^F_4_ (3d^8^4s^2^) ground state of Ni discourages significant bonding interaction, bonding in low-lying states of Ni_2_ results largely from the coupling of excited state Ni atoms. In particular, the lowest states of the Ni_2_ molecule can be expected to correlate with the ^3^D_3_ (3d^9^4s^1^) + ^3^D_3_ (3d^9^4s^1^) dissociation channel. At the generalized valence bond (GVB) and polarization CI (POL-CI) level of theory, (Upton and Goddard III, 1978) 30 of 45 low-lying states of Ni_2_, within the ^3^D_3_ + ^3^D_3_ dissociation channel, were found (Shim et al., 1979) to be singlets and triplets ordered energetically as

δδ (6 states) < πδ (8 states) < δσ (4 states) < ππ (6 states) < πσ (4 states) < σσ (2 states), with the six lowest states (of symmetries ^1^Γ_g_, ^1^Σ_g_^+^, ^3^Σ_g_^−^, ^1^Σ_u_^−^, ^3^Γ_u_, and ^3^Σ_u_^+^) being virtually degenerate and having an average equilibrium bond length, *R*_e_, of 2.04 Å, and binding energy, *D*_e_, of 2.29 eV. The designations δδ, πδ, et cetera, specify the positions of the holes in the dominant configurations of the 3d-orbitals at each atomic center which has a 3d^9^4s^1^ configuration (Shim et al., 1979). In brief, the correct energy spacing of the low-lying states of the Ni dimer is problematic for both theory and experiment.

The determination of energy ordering and spacing is no less complicated for the Ni atom, and provides insight into necessary levels of experimental and theoretical approaches. The ^3^F_4_ (3d^8^4s^2^) → ^3^D_3_ (3d^9^4s^1^) excitation energy has been determined by at least one experimental study (Moore, 1952) to be negative (-0.029 eV). This negative value was supported by *ab initio* wave function and density functional theory (DFT) calculations (Bauschlicher et al., 1982; Raghavachari and Trucks, 1989; Russo et al., 1994). A more recent study, (Schultz et al., 2005) that employed several functionals at all five rungs of Jacob’s ladder of DFT functionals, predicted the ground state configuration of the Ni atom as 3d^9^4s^1^ (^3^D_3_) with most of the functionals when using a triple-ζ quality basis set. On the other hand, multireference studies (Andersson and Roos, 1992; Murphy and Messmer, 1992) predicted a 3d^8^4s^2^ (^3^F_4_) ground state configuration for the Ni atom. Illustrating additional complexity, Upton and Goddard (Upton and Goddard III, 1978) found that averaging over *J* components (where *J* is the sum of spin and orbital angular momenta of the atom) of each state places the ^3^D_3_ (3d^9^4s^1^) state lower energetically than the ^3^F_4_ (3d^8^4s^2^) state.

One of the earliest theoretical studies (Cooper et al., 1972) on Ni_2_ employed the extended Hückel molecular orbital method and found a ^3^Σ_g_^-^ ground state with *R*_e_ = 2.21 Å, ω_e_ = 370 cm^−1^, and *D*_e_ = 2.45 eV, with dominant configuration 3dδ_g_^4^ 3dδ_u_^4^ 3dπ_u_^4^ 3dπ_g_^2^ 3dσ_g_^2^ 3dσ_u_^2^ 4sσ_g_^2^. On the other hand, the self-consistent field (SCF) scattered-wave (*X*_α_-sw) method (Rösch and Rhodin, 1974) found a ^1^Σ_g_^+^ ground state with configuration 3dδ_g_^4^ 3dδ_u_^4^ 3dπ_u_^4^ 3dπ_g_^4^ 3dσ_g_^2^ 4sσ_g_^2^; whereas the generalized valence bond (GVB) method (Melius et al., 1976) also predicted a ^1^Σ_g_^+^ ground state for Ni_2_, but with configuration 3dδ_g_^4^ 3dδ_u_^2^ 3dπ_u_^4^ 3dπ_g_^4^ 3dσ_g_^2^ 3dσ_u_^2^ 4sσ_g_^2^. A Hartree-Fock followed by limited CI study, (Shim et al., 1979) which explored a variety of states of Ni_2_ resulting from the ^3^F_4_ + ^3^F_4_ and ^3^D_3_ + ^3^D_3_ atomic combinations, found the ground state to be ^1^Σ_g_^+^ with the same configuration as was reported by the GVB study(Melius et al., 1976). The states ^1^Γ_g_ and ^1^Σ_u_^-^ were reported to be in close proximity to the ^1^Σ_g_^+^ state in the CI study. Other theoretical studies found the six δδ–hole states (i.e., ^1^Γ_g_, ^1^Σ_g_^+^, ^3^Σ_g_^-^, ^1^Σ_u_^-^, ^3^Γ_u_, ^3^Σ_u_^+^), resulting from the ^3^D_3_ + ^3^D_3_ atomic coupling, to be quasidegenerate(Basch et al., 1980; Noell et al., 1980; Wood et al., 1980).

Using an effective core potential basis set specifically optimized for the Ni atom in the ^3^D_3_ state within the generalized valence bond CI (GVBCI) method, Noell et al. (Noell et al., 1980) found the splitting of the six δδ–hole states of Ni_2_ to be quite small (≤0.1 eV), with the lowest states being ^1^Γ_g_ and ^1^Σ_g_^+^. Inclusion of polarization configurations involving single and double excitations to the virtual space (POLSDCI) placed the triplet states (^3^Σ_g_^-^, ^3^Γ_u_, ^3^Σ_u_^+^) approximately 0.07 eV below the singlets. At the singles and doubles CI (SDCI) level of theory, these authors found the energy splitting of the six lowest δδ–hole states of Ni_2_ to be less than 0.009 eV, with an average bond length of 2.26 Å and binding energy of 1.88 eV.

With a basis set similar to that used by Noell et al., (Noell et al., 1980) a contemporaneous restricted Hartree-Fock (RHF) and CI with single and double excitations (CISD) study predicted a ^3^Σ_u_^+^ ground state for Ni_2_, with spectroscopic data: *R*_e_ = 2.33 Å, ω_e_ = 211 cm^−1^, and *D*_e_ = 1.43 eV(Basch et al., 1980). Calculations by these authors at the same levels of theory using an all electron basis set corroborated the prediction of the ground state symmetry as ^3^Σ_u_^+^. On the other hand, a local spin density method (Harris and Jones, 1979) predicted a ^3^Σ_g_^-^ ground state with *R*_*e*_ = 2.18 Å, ω_e_ = 320 cm^−1^, and *D*_*e*_ = 2.70 eV. A slightly earlier CASSCF/CASPT2 study (Pou-Amérigo et al., 1994) that used an atomic natural orbital (ANO) type contraction of the (21s15p10d6f4g) primitive basis to [6s5p4d3f2g] for calculations without correlation of the semi-core 3s3p electrons, and a [10s9p8d3f2g] contracted basis for calculations involving the correlation of 3s3p electrons, likewise found the six lowest δδ–hole states of Ni_2_ to lie within a particularly narrow energy gap (0.04 eV) with the triplet states higher in energy than the singlets. However, after inclusion of scalar relativistic effects, the ground term was predicted as ^1^Γ_g_, with the ^1^Σ_g_^+^ term lying only 0.01 eV higher at the equilibrium geometry. Correlating the 3s3p electrons in these calculations predicted the ^1^Γ_g_ and ^1^Σ_g_^+^ states to be degenerate to the reported accuracy, with slightly improved spectroscopic constants relative to reference experimental values [i.e., *R*_e_ = 2.23 Å, *D*_e_ = 2.06 eV; and ω_e_ = 293 cm^−1^ for ^1^Γ_g_ versus ω_e_ = 294 cm^−1^ for ^1^Σ_g_^+^ compared with experimental values of *R*_e_ = 2.1545 ± 0.0004 Å, (Pinegar et al., 1995) *D*_*0*_ = 2.042 ± 0.002 eV, (Pinegar et al., 1995) and ω_e_ = 280 ± 20 cm^−1 ^(Ho et al., 1993)].

DFT studies of Ni_2_ have also been inconclusive. Yanagisawa et al. (Yanagisawa et al., 2000) used various DFT functionals to study the ^3^Σ_g_^-^ and ^3^Σ_u_^+^ states of Ni_2_ and found B3LYP (Becke, 1993) to predict the ^3^Σ_u_^+^ state to lie lower than ^3^Σ_g_^-^ whereas the rest of the functionals predicted the latter state to lie lower at the equilibrium geometry. However, the ^3^Σ_g_^-^ state that they found had a configuration that corresponded to the ππ–hole manifold rather than the δδ. Gutsev et al. (Gutsev and Bauschlicher, 2003) also found a ^3^Σ_g_^-^ ground state, with the same configuration as did Yanagisawa et al., (Yanagisawa et al., 2000) when using a variety of hybrid functionals. Contrarily, Diaconu et al. (Diaconu et al., 2004) found a singlet δδ–hole ground state (with a mixture of ^1^Γ_g_ and ^1^Σ_g_^+^) for Ni_2_ when using B3LYP with the (14s11p6d3f)/[8s6p4d1f] basis set, whereas use of the Stuttgart RSC ECP basis set (Dolg et al., 1987) with the same functional gave a triplet δδ–hole (with a mixture of ^3^Σ_g_^-^ and ^3^Γ_u_ symmetries) that lay 0.001 eV lower than the singlet δδ–hole state at the equilibrium geometry. Using functionals at all levels of Jacob’s ladder of DFT functionals, Schultz et al. (Schultz et al., 2005) also found different functionals to predict different ground state symmetries for Ni_2_, with all local spin density approximation (LSDA) functionals predicting a ^3^Σ_g_^-^ ground state and all generalized gradient approximation (GGA) and meta GGA functionals predicting a ^3^Π_u_ ground state; whereas hybrid GGA and hybrid meta GGA functionals found either ^3^Σ_u_^+^ or ^3^Σ_g_^-^ to lie lowest energetically. Du et al. (Du et al., 2008) used various functionals to study the low-lying states of Ni_2_. Their results that agreed best with experiment were obtained when using BLYP, which predicted a triplet σδ–hole (3d_z2_ σ_u_*^1^ 3d_x2-y2_ δ_u_*^1^) ground state. The space symmetry of this state was not reported. With the B3P86 functional, (Perdew, 1986; Becke, 1993) a quintet ground state was predicted for Ni_2_, (Shi-Ying and Zheng-He, 2008) although the space symmetry was not reported. The Perdew-Burke-Ernzerhof (PBE) exchange correlation functional (Perdew et al., 1996) predicted a ^3^Σ_g_^-^ ground state for Ni_2_, (Kamal et al., 2012) with spectroscopic constants *R*_e_ = 2.93 Å, *D*_e_ = 3.09 eV, and ω_e_ = 334.08 cm^−1^, which showed significant deviations from experimental values.

Some of the most recent wave function based calculations on Ni_2_ include those due to Dong et al. (Dong et al., 2013) using the symmetry-adapted-cluster configuration interaction (SAC-CI) method (Nakatsuji, 1979) and Cheskidov et al. (Cheskidov et al., 2012) using the average coupled pair functional (ACPF), (Gdanitz and Ahlrichs, 1988) average quadratic coupled cluster (AQCC), (Szalay and Bartlett, 1993) internally contracted single and double multireference configuration interaction (MRCI or MRCI with Davidson corrections, i.e., MRCI + Q), (Werner and Knowles, 1990) and N-electron valence state second-order perturbation theory (NEVPT2) (Angeli et al., 2001) methods. The study by Dong et al. (Dong et al., 2013) predicted a ^3^B_1u_ ground state (with *R*_e_ = 2.56 Å) for Ni_2_ in $D_{2h}$ symmetry which corresponds to ^3^Σ_u_^+^, ^3^Δ_u_ or ^3^Γ_u_ in $D_{\infty h}$. The study by Cheskidov et al. (Cheskidov et al., 2012) used the Dunning-type quadruple-ζ quality basis set, cc-pVQZ-DK (22s18p11d3f2g1h/[8s7p6d3f2g1h]), (Balabanov and Peterson, 2005) and found the ^1^Γ_g_ and ^1^Σ_g_^+^ δδ–hole states to be quasidegenerate for all five methods with the ^1^Σ_g_^+^ state lying lower when using AQCC, MRCI + Q, and MRCI methods and the two states fully degenerate (to the reported accuracy level) at the ACPF and NEVPT2 levels. At the ACPF level, the predicted ground state was instead ^1^Σ_u_^-^; inclusion of spin-orbit relativistic corrections within ACPF calculations led to an $0_{g}^{+}$ ground state (^1^Σ_g_^+^ + ^3^Σ_g_^-^ δδ–hole states), whereas the $0_{u}^{-}$ term (^1^Σ_u_^-^ + ^3^Σ_u_^+^ δδ–hole states) lay at only 0.009 ± 0.004 eV above the predicted ground state.

The above synopsis of previous work on Ni_2_ shows the difficulties involved in studying not only the spectroscopic constants, but even the ordering of the low-lying electronic states of Ni_2_. Although wave function methods generally support δδ–hole states (^1^Γ_g_, ^1^Σ_g_^+^, ^3^Σ_g_^-^, ^1^Σ_u_^-^, ^3^Γ_u_, ^3^Σ_u_^+^) as lying lowest energetically, the methods predict different ground state symmetries with some finding all six states to be degenerate. Similarly, experimental spectroscopic data have been obtained but most of the studies could not ascertain the ground state symmetry of the molecule. Our current study exploits the ability of the GVVPT2 method (Khait et al., 2002; Jiang et al., 2009) to describe well full PECs of ground- and excited-electronic states of complicated transition element dimers, such as has already been demonstrated on other problematic transition metal molecules [e.g., Cr_2_ and Y_2_ (Tamukong et al., 2012; Tamukong et al., 2014)]. It should be noted that of all previous theoretical work described above on electronic states of Ni_2_, only six of the articles reported full PECs of the states they investigated. Consequently, where other data is available, this study also provides further assessment of the capabilities of the GVVPT2 method for difficult transition metal dimers, including the bond breaking regions. We have constructed full PECs of 21 states of Ni_2_. All (nonrelativistic) calculations used the Dunning-type cc-pVTZ basis set (Balabanov and Peterson, 2005), and calculations were performed using $D_{2h}$ symmetry. Furthermore, low-lying ^1^Γ_g_ and ^1^Σ_g_^+^ states were further studied with scalar relativistic effects included in GVVPT2 (Tamukong et al., 2014) through the spin-free exact two component (sf-X2C) method (Liu, 2010; Cheng and Gauss, 2011; Li et al., 2012; Li et al., 2013). Relativistic calculations used the cc-pVTZ-DK basis set (Balabanov and Peterson, 2005). The rest of the paper is organized as follows: *Methods* section briefly reviews key features of GVVPT2 and the spin-free exact two component (sf-X2C) methods, and describes computational details; the results are presented and discussed in *Results and Discussion* section; while conclusions are drawn in *Conclusion* section.

## Methods

### GVVPT2

The GVVPT2 method for electron correlation in molecules has been thoroughly described elsewhere, (Khait et al., 2002; Jiang et al., 2009) as has its application to some challenging systems (Mbote et al., 2010; Khait et al., 2012; Tamukong et al., 2012; Mokambe et al., 2013; Tamukong et al., 2014). Here, salient features of the GVVPT2 method relevant to the present study are reviewed. In GVVPT, the total Hilbert space of many-electron functions [e.g., configuration state functions (CSFs)] with appropriate molecular space and spin symmetry (*L*) is partitioned into a model space (*L*_M_), and an external space (*L*_Q_) whose electronic configurations are derived from configurations generated from the model space by single and double electron excitations into virtual orbitals, *L* = *L*_M_ ⊕ *L*_Q_. The model space is further partitioned into a primary subspace, spanned by a set of reference functions that are linear combinations of CSFs (typically the lowest MCSCF or CASSCF states), and a secondary subspace. States within the primary subspace are then perturbatively corrected through primary-external (P–Q) interactions whereas the secondary subspace serves as a buffer energetically separating the primary and external subspaces. This energy buffer circumvents most intruder state problems that plague many multireference perturbation theory techniques. An effective Hamiltonian matrix $\left(H_{\mathrm{MM}}^{\mathrm{eff}}\right)$ is constructed with the same dimension as the model space, and its diagonalization includes both perturbatively corrected primary and unperturbed secondary subspace states. To guarantee continuity and smoothness of CSF responses (and ultimately PECs) even in situations of quasidegeneracies between primary and external states, GVVPT2 uses a continuously varying nonlinear denominator shift that arises from a resolvent that is both degeneracy-corrected and contains a hyperbolic tangent function as a switching function from nondegenerate to degenerate regimes (Khait et al., 2002). This resolvent can be written

$$X_{qi}=\;\frac{\tanh\left(\Delta_{i}\right)}{\Delta_{i}}\;H_{qi}=\;\frac{\tanh\left(\Delta_{i}\right)}{\Delta_{i}}\;\sum_{m\in L_{M}}H_{qm}C_{mi}$$ (1a)

$$\Delta_{i}=\frac{1}{2}\left(\varepsilon_{q}^{i}-\varepsilon_{i}^{\left(0\right)}\right)+\;\frac{1}{2}\;\sqrt{{\left(\varepsilon_{q}^{i}-\varepsilon_{i}^{\left(0\right)}\right)}^{2}+4\sum_{q\in e}H_{qi}^{2}}$$ (1b)

where the C_*mi*_ denote components of eigenvectors of the unperturbed model Hamiltonian; $\varepsilon_{i}^{\left(0\right)}$ is the reference Møller-Plesset-type energy while $\varepsilon_{q}^{\left(0\right)}$ is the state-specific zeroth-order energy of external CSF *q*. As with all studies of specific molecules with GVVPT2 since ca. 2005, semicanonical orbitals from the MCSCF are used. The mathematical robustness of GVVPT2 allows it to support both complete and incomplete model spaces. By construction, GVVPT2 is subspace-specific (N.B. excited states of the same symmetry as lower-lying states can be calculated) and is spin-adapted.

### Spin-Free Exact Two Component Method

Matrix formulations of two-component relativistic methods have had significant successes in molecular electronic structure calculations, following Dyall’s seminal Normalized Elimination of Small Component (NESC) paper (Dyall, 1997) in 1997. For a summary of development of matrix two-component methods, we refer the reader to Ref. (Liu, 2014). In this work, we adopt the spin-free version of exact two component (sf-X2C) (Liu, 2010; Cheng and Gauss, 2011; Li et al., 2012; Li et al., 2013) that was previously used with GVVPT2 (Tamukong et al., 2014). The following Hamiltonian, written in second quantization form, incorporates scalar relativistic effects through the sf-X2C approach

$$H=\sum_{pq}{\left[h_{+,\mathrm{sf}}^{\mathrm{X2C}}\right]}_{pq}a_{p}^{+}a_{q}\;+\;\frac{1}{2}\;\sum_{pqrs}\left(pr|qs\right)a_{p}^{+}a_{q}^{+}a_{s}a_{r}$$ (2)

where the first term is the one-electron spin-free part of the exact two-component (X2C) Hamiltonian, while the second is the unmodified Coulombic two-electron term. The sf-X2C Hamiltonian for positive energy states derives from a modified Dirac Hamiltonian, **h** ^D^ which is decomposed into spin-free (sf) and spin-dependent (sd) parts.

$$h^{D}=\;h_{\mathrm{sf}}^{D}+h_{\mathrm{sd}}^{D}=\;\left(\begin{array}{cc} V & T\\ T & \frac{\alpha^{2}}{4}W_{\mathrm{sf}}-T \end{array}\right)+\;\left(\begin{array}{cc} 0 & 0\\ 0 & \frac{\alpha^{2}}{4}W_{\mathrm{sd}} \end{array}\right)$$ (3)

where **V** is the matrix representation of the external nuclear attraction potential operator; **T** is the matrix representation of the kinetic energy operator; α is the fine-structure constant; while **W** is the matrix representation of the operator

$$W=\;\left(\vec{\sigma }\;\cdot \;\vec{p}\right)V\;\left(\vec{\sigma }\;\cdot \;\vec{p}\right)=\;\;\vec{p}\;\cdot V\;\vec{p}+i\;\vec{\sigma }\;\cdot \;\left(\vec{p}\;V\;\times \;\vec{p}\right)=\;W_{\mathrm{sf}}+\;W_{\mathrm{sd}}$$ (4)

The spin-free (sf) part of W, that is W_sf_, describes scalar relativistic effects whereas the spin-dependent (sd) part, W_sd_, incorporates spin-orbit coupling effects.

### Computational Details

Unperturbed wavefunctions for GVVPT2 calculations were obtained from multiconfigurational self-consistent field (MCSCF) reference functions, using the local code “undmol.” The macroconfiguration approach (Khait et al., 2004) was used in all MCSCF and GVVPT2 calculations. In the macroconfiguration scheme, active orbitals are partitioned into orbital groups based on physical and mathematical intuition, such as orbital type or energy, and electrons are assigned to active orbital groups. Each unique assignment of electrons to active orbital groups defines a macroconfiguration, κ(**n**). Orbital rotations within each active orbital group are redundant (in MCSCF calculations), as all possible distributions of electrons within each group leading to the desired total spin and space symmetry are allowed. Each κ(**n**) generates a unique set of configurations, and hence configuration state functions (CSFs) that are orthogonal to those of all other κ(**n**). Additionally, the macroconfiguration approach permits a large number of noninteracting electronic configuration pairs to be efficiently screened by recognizing that all matrix elements resulting from configurations that differ by more than two electrons must be identically zero. The macroconfiguration formalism also provides an efficient way of generating excited configurations.

**FIGURE 1:**
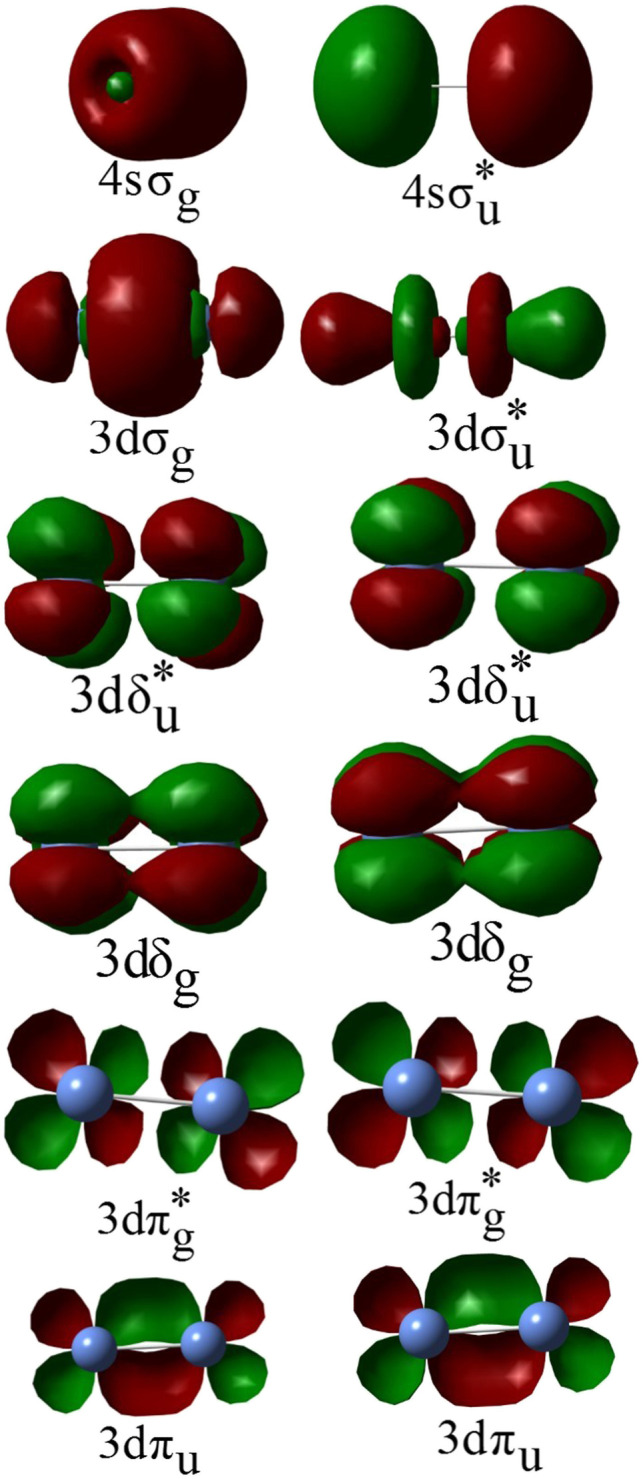
Example of MCSCF 3d- and 4s-derived molecular orbitals of the Ni_2_ molecule.

In all calculations, the active space consisted of 3d and 4s-derived molecular orbitals (MOs) of Ni_2_ (Figure 1 shows representative 3d and 4s-derived MOs that were used to constitute the active spaces in this study). Depending on the specific state being investigated, some of the 3d-derived MOs and/or 4s-derived MOs were restricted to be doubly occupied in the MCSCF calculations, but were included with the 3s- and 3p-derived MOs in the active core and correlated at the GVVPT2 level of theory. For example, in all MCSCF calculations of δδ–hole states the 3dσ and 3dπ MOs were placed in the active core and only correlated at the GVVPT2 level. Similarly, the 3dσ and 3dπ electrons were kept doubly occupied in MCSCF calculations of ππ–hole states while only the 3dσ electrons were kept doubly occupied in MCSCF calculations of δπ–hole states, whereas the 4sσ, or 4sσ + 3dπ, or 4sσ + 3dσ orbitals were kept doubly occupied in MCSCF calculations of states within the ^3^F_4_ (3d^8^4s^2^) + ^3^F_4_ (3d^8^4s^2^) dissociation channel. The remaining orbitals in the active space were partitioned into sets (or so-called orbital groups), leading to configurations that describe δδ–, δπ–, δσ–, ππ–, πσ–, or σσ–hole states from the ^3^D_3_ (3d^9^4s^1^) + ^3^D_3_ (3d^9^4s^1^) atomic combination or configurations that describe ^3^D_3_(3d^9^4s^1^) +^3^F_4_(3d^8^4s^2^) and ^3^F_4_(3d^8^4s^2^) + ^3^F_4_(3d^8^4s^2^) atomic couplings. In the present study, we indicate the positions of holes within the subspace of active 3d–derived MOs by specifying the active orbital types that qualitatively describe where holes exist (e.g., δδ–, δπ–, ππ–) as did Shim et al. (Shim et al., 1979) and Noel et al. (Noell et al., 1980)

All δδ–states were computed using a single reference macroconfiguration,

$$\kappa_{1}\left(n\right)={\left(3d\delta_{g}3d\delta_{u}^{*}3d\delta_{g}3d\delta_{u}^{*}\right)}^{6}{\left(4s\sigma_{g}4s\sigma_{u}^{*}\right)}^{2}$$ (5)

where the superscripts denote the number of electrons in each orbital group. The semi-core 3s3p electrons were correlated together with those derived from 3d_z2,_ 3d_xz_ and 3d_yz_ at the GVVPT2 level. For four of the computed δδ–states, additional calculations were also performed in which the 3s3p were kept doubly occupied throughout (i.e., at both the MCSCF and GVVPT2 levels). When using the cc-pVTZ basis set, (Balabanov and Peterson, 2005) reference κ_1_(**n**) generated 8 model and 27,891,120 total CSFs (in D_2h_ symmetry) for the 1 ^3^Σ_u_^+^ and 1 ^3^Γ_u_ states; 8 model and 15,290,666 total space CSFs for the 1^1^Σ_u_^−^, 1^1^Σ_g_^−^, and 1^1^Γ_u_ states; 10 model and 27,982,592 all space CSFs for the 1^3^Σ_g_^-^ and 1^3^Σ_u_^-^ states; and 12 model versus 15,270,687 all space CSFs for the X ^1^Γ_g_ and 1 ^1^Σ_g_^+^ states. Without correlating the 3s3p electrons at the GVVPT2 level, the dimensions were: 12 model space CSFs versus 3,593,707 total CSFs for the X ^1^Γ_g_ and 1 ^1^Σ_g_^+^ states; and 8 model versus 6,434,550 all space CSFs for the 1^3^Σ_u_^+^ and 1^3^Γ_u_ states. Relativistic calculations on the X ^1^Γ_g_ and 1 ^1^Σ_g_^+^ states utilized the same reference macroconfiguration, κ_1_(**n**). A δδ ^3^Γ_u_ state was computed with 4 active electrons in 4 orbitals using reference

$$\kappa_{2}\left(n\right)={\left(3d_{x^{2}-y^{2}}\delta_{g}3d_{x^{2}-y^{2}}\delta_{u}^{*}\right)}^{6}{\left(4s\sigma_{g}4s\sigma_{u}^{*}\right)}^{2}$$ (6)

which gave rise to 4 model space and 7,518,688 all space CSFs, using the cc-pVTZ basis set. The ππ–states were computed using reference

$$\kappa_{3}\left(n\right)={\left(3d\pi_{u}3d\pi_{g}^{*}3d\pi_{u}3d\pi_{g}^{*}\right)}^{6}{\left(4s\sigma_{g}\;4s\sigma_{u}^{*}\right)}^{2}$$ (7)

which is similar to κ_1_(**n**) but with delta replaced with pi orbitals. This macroconfiguration gave rise to 12 model space and 15,267,629 all space CSFs for the 1^1^Δ_g_ and 2^1^Σ_g_^+^ states when using the cc-pVTZ basis set and D_2h_ molecular space symmetry. The δπ–states were computed from

$$\kappa_{4}\left(n\right)=\;{\left(3d\delta_{g}3d\delta_{u}^{*}3d\delta_{g}3d\delta_{u}^{*}\right)}^{7}{\left(3d\pi_{u}3d\pi_{g}^{*}3d\pi_{u}3d\pi_{g}^{*}\right)}^{7}{\left(4s\sigma_{g}4s\sigma_{u}^{*}\right)}^{2}$$ (8)

κ_4_(**n**) generated 16 model space and 27,178,852 total space CSFs for the 1^1^Φ_g_ and 1^1^Π_g_ states versus 20 model and 50,736,846 all space CSFs for the 1^3^Φ_g_ and 1^3^Π_g_ states, when using the cc-pVTZ basis set.

Within the ^3^F_4_ + ^3^F_4_ manifold, the 3^1^Σ_g_^+^, 1^3^Σ_g_^+^, 2 ^3^Σ_g_^+^, 1^3^Δ_u_, and 2 ^3^Σ_u_^+^ δπ–states were computed using

$$\kappa_{5}\left(n\right)=\;{\left(3d\delta_{g}3d\delta_{u}^{*}3d\delta_{g}3d\delta_{u}^{*}\right)}^{6}{\left(3d\pi_{u}3d\pi_{g}^{*}3d\pi_{u}3d\pi_{g}^{*}\right)}^{6}$$ (9)

In these calculations, the 3s, 3p, 3d_z2_, and 4s electrons were kept doubly occupied at the MCSCF level but correlated at the GVVPT2 level of theory. Reference κ_5_(**n**) resulted in 40 model versus 55,053,638 total CSFs for the 3^1^Σ_g_^+^ state; 36 model and 103,306,512 all space CSFs for the 1^3^Σ_g_^+^ and 2 ^3^Σ_g_^+^ states; and 40 model versus 103,312,902 all space CSFs for the 1^3^Δ_u_ and 2 ^3^Σ_u_^+^ states when using the cc-pVTZ basis set.

Two δπ–states, 2 ^1^Γ_g_ and 2 ^1^Δ_g_, were computed within the ^3^F_4_ + ^3^F_4_ manifold using

$$\kappa_{6}\left(n\right)=\;{\left(3d\delta_{g}3d\delta_{u}^{*}3d\delta_{g}3d\delta_{u}^{*}\right)}^{6}{\left(3d\sigma_{g}3d\sigma_{u}^{*}\right)}^{2}$$ (10)

Reference κ_6_(**n**) generated 12 model space and 15,270,687 all space CSFs for the computed states using the cc-pVTZ basis set. Two quintet δπσ–states (i.e., 1^5^Φ_u_ and 1^5^Π_u_) were also computed within the ^3^F_4_ + ^3^F_4_ manifold, using

$$\kappa_{7}\left(n\right)=\;{\left(3d\delta_{g}3d\delta_{u}^{*}3d\delta_{g}3d\delta_{u}^{*}\right)}^{6}{\left(3d\pi_{u}3d\pi_{g}^{*}3d\pi_{u}3d\pi_{g}^{*}\right)}^{7}{\left(3d\sigma_{g}3d\sigma_{u}^{*}\right)}^{3}$$ (11)

This reference, κ_7_(**n**), led to 12 model versus 69,738,914 total CSFs for the computed quintet states. Lastly, a limited study was done on two quintet states within the ^3^D_3_(3d^9^4s^1^) + ^3^F_4_(3d^8^4s^2^) manifold at short bond lengths. While reference

$$\kappa_{8}\left(n\right)=\;{\left(3d\delta_{g}3d\delta_{u}^{*}3d\delta_{g}3d\delta_{u}^{*}\right)}^{7}{\left(3d\pi_{u}3d\pi_{g}^{*}3d\pi_{u}3d\pi_{g}^{*}\right)}^{6}{\left(4s\sigma_{g}4s\sigma_{u}^{*}\right)}^{3}$$ (12)

can describe the 1^5^Δ_g_ and 2 ^5^Δ_g_ δπσ–states resulting from the ^3^D_3_(3d^9^4s^1^) + ^3^F_4_(3d^8^4s^2^) manifold of molecular states adequately at short internuclear distances, it cannot be expected to do so at long internuclear distances and our studies were restricted to the shorter bond lengths for these two states. Reference κ_8_(**n**) generated 12 model CSFs versus 69,740,135 total space CSFs.

The reference macroconfigurations described above were used to define the active space, while all lower energy MOs were doubly occupied in MCSCF calculations. Initial MOs for MCSCF calculations were obtained from approximate natural orbitals of second-order restricted Møller−Plesset perturbation (RMP2) calculations from a closed-shell Hartree−Fock (HF) reference. At the GVVPT2 level, 3s, 3p, and all 3d and/or 4s electrons were correlated whether they were or were not at the MCSCF level. For comparison purposes, a few of the GVVPT2 calculations were performed without correlating the 3s and 3p electrons. Calculations that accounted for scalar relativistic effects employed the sf-X2C method described above. Finally, to aid in interpretation, the effective bond order (EBO) was computed, and used the following expression

$$\eta =\;\frac{\sum_{i}\chi_{i}c_{i}^{2}}{\sum_{i}c_{i}^{2}}$$ (13)

where *η* is the EBO, *χ*_*i*_ is the EBO for the *i*th CSF, while *c*_*i*_^*2*^ is its corresponding weight. For each CSF used to estimate EBO, χ_i_ was determined as

$$\chi_{i}=\;\frac{1}{2\;}\left(n_{b}-\;n_{\mathrm{ab}}\right)$$ (14)

where *n*_b_ and *n*_ab_ are the numbers of bonding and antibonding electrons, respectively. Vibrational frequencies were obtained by 3-point finite differencing near the minima of the curves.

## Results and Discussion

Where indicated, the letter “R” in parentheses following a molecular term denotes that scalar relativistic effects were included in the calculations, while the expression “no 3s3p” in parentheses after a molecular term symbol denotes that 3s and 3p electrons were not correlated in GVVPT2 calculations.

### The δδ–Hole States

**FIGURE 2:**
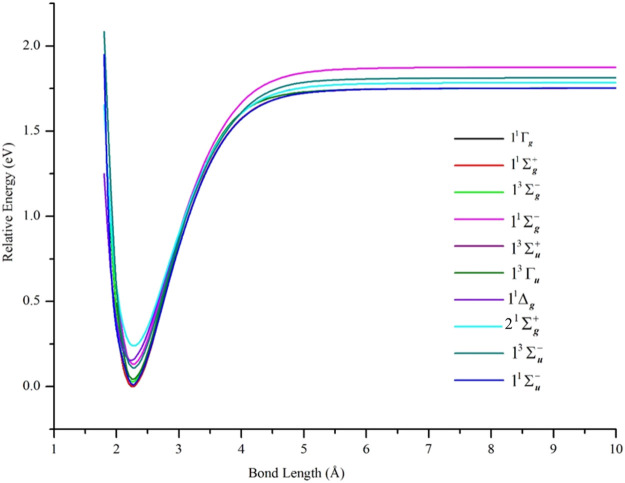
PECs of low-lying electronic states of Ni_2_ computed at the GVVPT2 level of theory using the cc-pVTZ basis set. All energies are plotted relative to the lowest energy value of the ground X ^1^Γ_g_ term. For all states, the holes are in the 3d delta orbitals (δδ–holes) except for 1^1^Δ_g_ and 2 ^1^Σ_g_^+^ states which are ππ–hole states.

PECs of the δδ–hole states are shown in Figure 2 and the data describing them are in Table 1. In agreement with results from other high level *ab initio* methods, the lowest states of Ni_2_ were found to be δδ–hole states of the ^3^D_3_ (3d^9^4s^1^) + ^3^D_3_ (3d^9^4s^1^) manifold. In particular, the ground state was found to be X ^1^Γ_g_, with the 1^1^Σ_g_^+^ state lying only 16.40 cm^−1^ (0.002 eV) higher at the equilibrium geometry. After including scalar relativistic effects, the energy gap between these states slightly increased to 23.39 cm^−1^ at equilibrium, with the X ^1^Γ_g_ term having spectroscopic constants: *R*_e_ = 2.20 Å, *D*_e_ = 1.95 eV, and ω_e_ = 296 cm^−1^. These results are in good agreement with experimental data (*R*_e_ = 2.1545 ± 0.0004 Å, (Cooper et al., 1972) $D_{0}^{o}$ = 2.042 ± 0.002 eV, (Cooper et al., 1972) and ω_e_ = 280 ± 20 cm^−1^) (Rösch and Rhodin 1974) and with the relativistic CASSCF/CASPT2 results of Pou‐Amérigo et al. (Pou‐Amérigo et al., 1994) who also found the lowest ^1^Γ_g_ and ^1^Σ_g_^+^ terms to be quasidegenerate (with *R*_e_ = 2.23 Å, *D*_e_ = 2.06 eV, and ω_e_ = 293 cm^−1^ for the ^1^Γ_g_ term). These results are also in good agreement with the time-delayed resonant two-photon ionization study of Morse and co-workers (Pinegar et al., 1995) that predicted either a ^3^Γ_u_ or ^1^Γ_g_ state as the ground state of Ni_2_ and the scalar relativistic calculations of Cheskidov et al. (Cheskidov et al., 2012) who found the lowest ^1^Γ_g_ and ^1^Σ_g_^+^ terms to be degenerate at the ACPF and NEVPT2 levels of theory and the ^1^Σ_g_^+^ term to lie very slightly lower than the ^1^Γ_g_ at the AQCC, MRCI, and MRCI + Q levels of theory. At 2.25 Å, we found the EBO to be 0.963 and 0.960 for the ^1^Γ_g_ and ^1^Σ_g_^+^ states, respectively (using the largest dozen CSFs). In relativistic GVVPT2 calculations, these EBOs increased slightly to 0.975 for ^1^Γ_g_ and 0.972 for ^1^Σ_g_^+^ at this geometry. The major configurations describing the X ^1^Γ_g_ and 1^1^Σ_g_^+^ states involved two holes in the same δ–type; i.e., 0.445 × (3d_xy_δ_g_^2^ 3d_xy_δ_u_^∗2^ 3d_x2-y2_δ_g_^2^ 4sσ_g_^2^ + 3d_xy_δ_g_^2^ 3d_x2-y2_δ_g_^2^ 3d_x2-y2_δ_u_^∗2^ 4sσ_g_^2^) and 0.077 × (3d_xy_δ_g_^2^ 3d_xy_δ_u_^∗2^ 3d_x2-y2_δ_u_^∗2^ 4sσ_u_^∗2^ - 3d_xy_δ_u_^∗2^ 3d_x2-y2_δ_g_^2^ 3d_x2-y2_δ_u_^∗2^ 4sσ_u_^∗2^), where coefficients are amplitudes and each term within parentheses denotes active orbitals of each leading CSF of the GVVPT2 wavefunction.

**TABLE 1:** Equilibrium bond lengths, *R*_e_ (Å), binding energies, *D*_e_ (eV), harmonic frequencies, ω_e_ (cm^−1^), and adiabatic transition energies, *T*_e_ (cm^−1^), of electronic states of Ni_2_ calculated at the GVVPT2 level of theory using the cc-pVTZ basis set (or cc-pVTZ-DK for scalar relativistic calculations).

Molecular term	*R*_e_ (Å)	*D*_e_ (eV)	ω_e_ (cm^−1^)	*T*_e_ (cm^−1^)
δδ–hole states				
Computed using κ_1_(**n**)				
X ^1^Γ_g_	2.26	1.75	276.0	
X ^1^Γ_g_ (no 3s3p)	2.27	1.66	268.5	
X ^1^Γ_g_ (R)	2.20	1.95	296.0	
Experiment	2.1545 ± 0.0004^a^	2.042 ± 0.002^a^	280 ± 20^b^	
1^1^Σ_g_^+^	2.26	1.75	276.8	16.40
1^1^Σ_g_^+^ (R)	2.20	1.95	297.0	23.39
1^1^Σ_g_^+^ (no 3s3p)	2.28	1.65	263.3	16.56
1^1^Σ_u_^-^	2.27	1.74	274.2	91.09
1^3^Σ_u_^+^	2.27	1.71	274.9	349.60
1^3^Σ_u_^+^ (no 3s3p)	2.28	1.62	267.4	309.58
1^3^Γ_u_	2.27	1.71	274.9	351.11
1^3^Γ_u_ (no 3s3p)	2.28	1.62	267.4	310.31
1^3^Σ_g_^-^	2.26	1.72	275.0	221.98
1^3^Σ_u_^-^	2.27	1.70	273.9	882.59
1^1^Σ_g_^-^	2.27	1.74	270.2	1058.87
2 ^1^Σ_u_^-^	2.75	0.11	73.5	18575.76
Computed using κ_2_(**n**)				
2 ^3^Γ_u_	2.27	1.71	275.0	2442.21

a Ref. (Cooper et al., 1972) (the reported binding energy is for D_0_).

b Ref. (Rösch and Rhodin, 1974).

**FIGURE 3:**
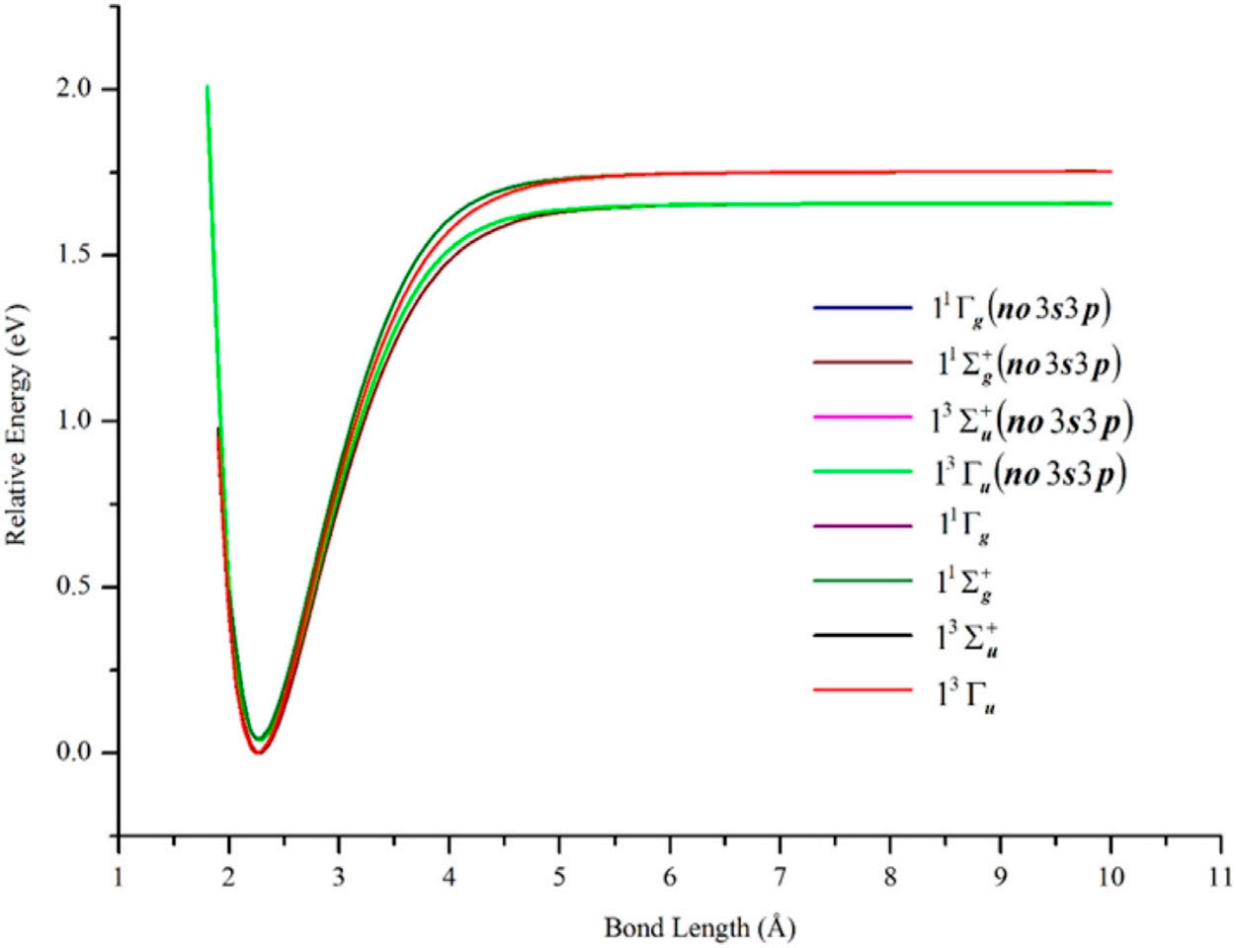
PECs of low-lying δδ–hole electronic states of Ni_2_ computed at the GVVPT2 level of theory, with and without the correlation of 3s3p semi-core electrons, using the cc-pVTZ basis set. All energies are plotted relative to the lowest energy value of the ground X ^1^Γ_g_ term.

**FIGURE 4:**
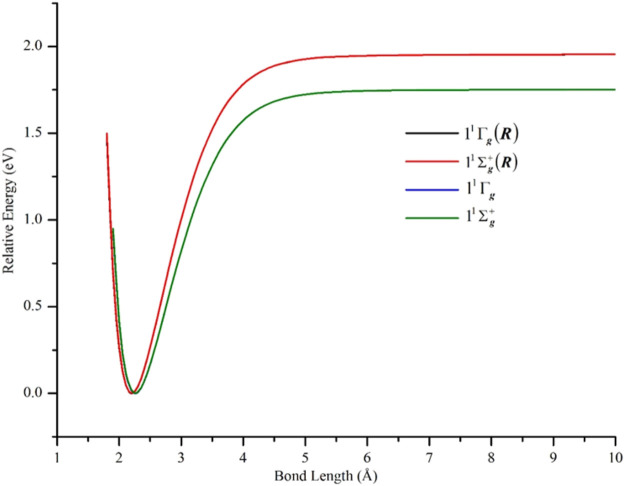
PECs of the lowest-lying δδ–hole X ^1^Γ_g_ and 1^1^Σ_g_^+^ states of Ni_2_ computed at the GVVPT2 level of theory, with and without scalar relativity included, using the cc-pVTZ (or cc-pVTZ-DK) basis set. Non-relativistic energies are plotted relative to the lowest energy value of the ground X ^1^Γ_g_ term while relativistic energies are plotted relative to the lowest energy of the X ^1^Γ_g_ (R) term.

The semi-core 3s3p electrons were found to be important in the description of low-lying states of Ni_2_. The inclusion of 3s3p electron correlation at the GVVPT2 level increased the binding energies by 0.09 eV for X ^1^Γ_g_, 0.10 eV for 1^1^Σ_g_^+^, and 0.09 eV for 1^3^Σ_u_^+^ and 1^3^Γ_u_ states in non-relativistic calculations. As can be seen in Figure 3 and Table 1, the effects of the 3s3p electrons on the equilibrium bond lengths and harmonic frequencies for these states are minimal whereas inclusion of such core-valence correlation raises, for example, the binding energy of X ^1^Γ_g_ from 1.66 to 1.75 eV compared to a reference *D*_*0*_ value of 2.042 ± 0.002 eV (Cooper et al., 1972). Scalar relativistic effects shortened the bond length of X ^1^Γ_g_ by 0.06 Å and further increased the bond energy by 0.20 to 1.95 eV which agreed even better with the reference experimental values (see Figure 4 and Table 1). The 3s3p electrons did not have any effect on the EBOs of X ^1^Γ_g_ and 1^1^Σ_g_^+^; the EBOs were determined as 0.962 and 0.959 at 2.27 Å for X ^1^Γ_g_ and 1^1^Σ_g_^+^, respectively, when the 3s3p electrons were not correlated compared to 0.963 vs. 0.960 when the semi-core electrons (3s3p) were correlated. Note the quasidegeneracy in the X ^1^Γ_g_ and 1^1^Σ_g_^+^ states. For example, in Figure 4, the blue and green curves for the X ^1^Γ_g_ and 1^1^Σ_g_^+^ states, respectively, lie on top of each other (only the green is visible). Also, the black and red curves for the X ^1^Γ_g_ (R) and 1^1^Σ_g_^+^ (R) states lie on each other (only the red curve is visible).

The 1^1^Σ_u_^-^ state, which was predicted as the ground state of Ni_2_ at the ACPF level of theory (Cheskidov et al., 2012) and found to lie quite close to a ^1^Σ_g_^+^ ground state in a limited CI study (Ahmed and Nixon, 1979), was found at the GVVPT2 level to lie 91.09 cm^−1^ above the X ^1^Γ_g_ state at equilibrium. The 2^1^Σ_u_^-^ state, however, lay much higher energetically (18,575.76 cm^−1^ above the ground state at equilibrium).

As can be seen in Table 1, GVVPT2 predicted the triplet δδ–hole states, 1^3^Σ_g_^-^, 1^3^Σ_u_^+^, and 1^3^Γ_u_, to lie energetically in the order 1^3^Σ_g_^-^< 1^3^Σ_u_^+^ < 1^3^Γ_u_. Cheskidov et al. (Cheskidov et al., 2012) found this same ordering at the ACPF, AQCC, MRCI and MRCI + Q levels of theory, whereas their NEVPT2 calculations predicted the ordering 1^3^Σ_u_^+^ < 1^3^Γ_u_ < 1^3^Σ_g_^-^, with the 1^3^Σ_g_^-^ state lying at least 139 cm^−1^ higher than the other two states. It should be noted that the vertical excitation energies in Ref. (Cheskidov et al., 2012) were not determined at the equilibrium geometries of the computed states. The 1^3^Σ_u_^+^ state, which was predicted as the ground state of Ni_2_ in some previous wavefunction (Wood et al., 1980; Schultz et al., 2005) and DFT (Diaconu et al., 2004; Kramida et al., 2013) studies, was found in our current study to lie 349.60 cm^−1^ above the X ^1^Γ_g_ state at the equilibrium geometry. Likewise, the 1^3^Σ_g_^-^ state reported in other studies (Murphy and Messmer, 1992; Diaconu et al., 2004; Kramida et al., 2013) as the ground state of Ni_2_ lay 221.98 cm^−1^ higher at equilibrium. The 1^3^Σ_u_^-^ state was found to have a bond length and bond energy comparable to those of 1^3^Σ_g_^-^, 1^3^Σ_u_^+^, and 1^3^Γ_u_ but lying at least 531.48 cm^−1^ higher in energy. The EBOs for these triplet states were 0.971 for 1^3^Σ_g_^-^, 0.933 for 1^3^Σ_u_^+^ and 1^3^Γ_u_, and 0.923 for 1^3^Σ_u_^-^ in the vicinity of their equilibrium geometries (again computed with 10 leading CSFs). The major configurations for the δδ–hole triplet states involved a doubly occupied 4sσ_g_ bonding orbital.

The 1^3^Γ_u_ state, in which both δ–holes were in the 3d_x2-y2_ δ_g_ and δ_u_^∗^ orbitals, was computed using reference κ_2_(**n**). As can be seen in Table 1, this state was found to have spectroscopic constants comparable to other δδ–hole triplet states but lay much higher energetically (2442.21 cm^−1^ above the ground state at equilibrium). The present results suggest that the 3dδ orbitals are indeed split in the bonding interaction. Since they are nondegenerate, the Aufbau principle suggests that energetically low-lying orbitals (the bonding 3dδ orbitals) be occupied before higher ones. Moreover, Hund’s rule suggests that orbitals with similar energies (in this case, 3dδ_u_^∗^ orbitals) be singly occupied before electron pairing occurs. This seems to bring qualitative understanding into why the 1^3^Σ_g_^-^, 1^3^Σ_u_^+^, and 1^3^Γ_u_ states in which the 3d_x2-y2_δ_u_^∗^ and 3d_xy_δ_u_^∗^ are singly occupied lie energetically lower than the 2 ^3^Γ_u_ state for which the 3d_x2-y2_δ_g_ and 3d_x2-y2_δ_u_^∗^ are singly occupied.

### The δπ–Hole and ππ–Hole States

**FIGURE 5:**
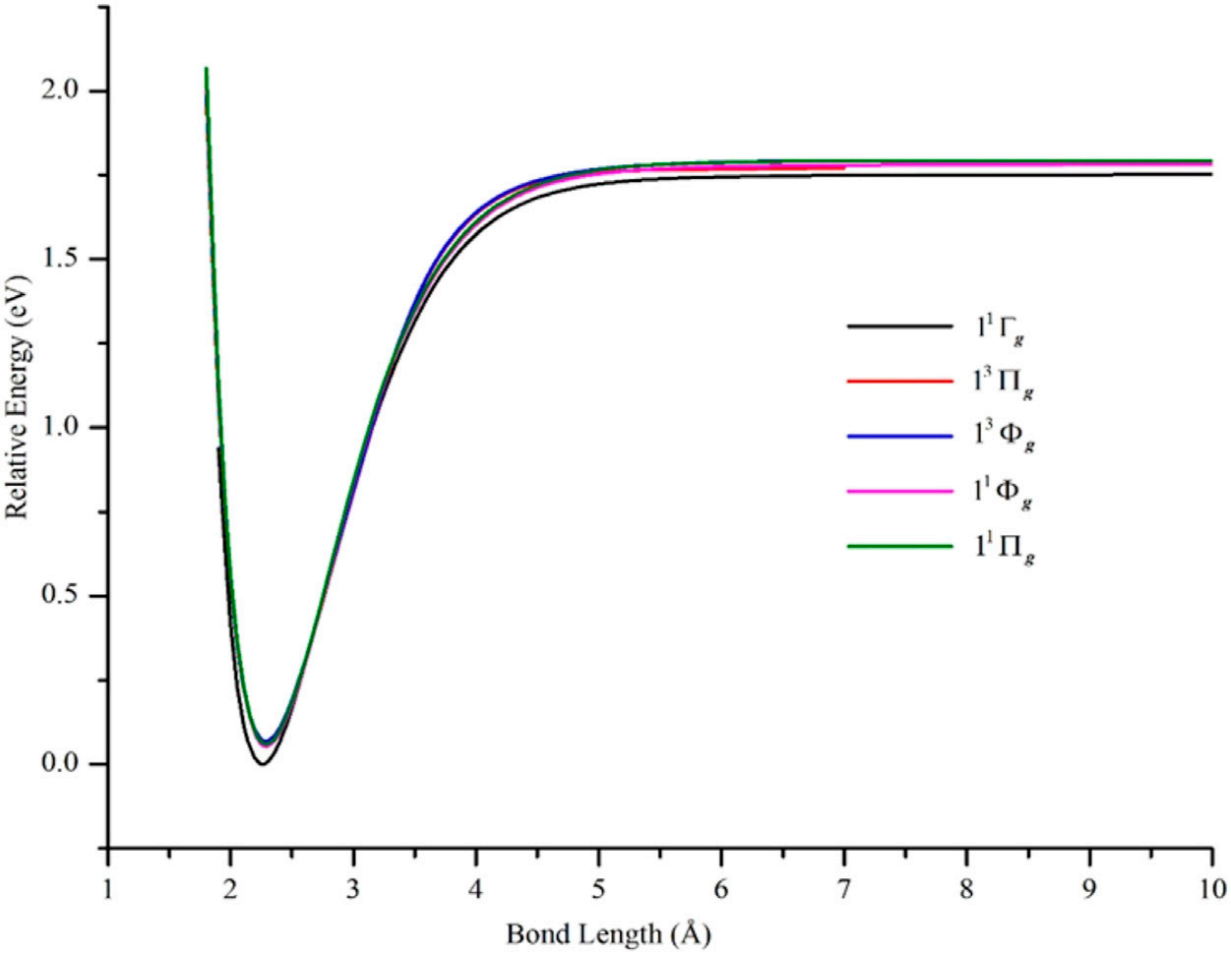
PECs of low-lying δπ–hole electronic states of Ni_2_ computed at the GVVPT2 level of theory using the cc-pVTZ basis set compared with the PEC of the X ^1^Γ_g_ ground term. All energies are plotted relative to the lowest energy value of the X ^1^Γ_g_ ground term.

The PECs of the computed ππ–hole states (1^1^Δ_g_ and 2^1^Σ_g_^+^) are shown in Figure 2, while those for the δπ–hole states (1^1^Φ_g_, 1^1^Π_g_, 1^3^Π_g_, and 1^3^Φ_g_) are shown in Figure 5 and compared with the ground state PEC. The data describing these curves are in Table 2. GVVPT2 predicted the ππ–hole states to lie higher in energy than the δπ–hole states, in agreement with previous studies (Ahmed and Nixon, 1979; Rasanen et al., 1987; Russo et al., 1994). For all four δπ–hole states studied, the major CSFs involved a doubly occupied 4sσ_g_ bonding orbital. Thus, the main configurations of the 1^1^Φ_g_ and 1^1^Π_g_ states involved an unpaired spin-increasing electron in the 3dδ subspace and an unpaired spin-decreasing electron in the 3dπ subspace, e.g., 3dδ_g_^2^ 3dδ_u_^∗2^ 3dδ_g_^2^ 3dδ_u_^∗(↑)^ 3dπ_u_^2^ 3dπ_g_^∗2^ 3dπ_u_^2^ 3dπ_g_^∗(↓)^ 4sσ_g_^2^, whereas the major configurations of the 1^3^Φ_g_ and 1^3^Π_g_ states were similar to those of the corresponding singlet states but with two unpaired spin-increasing electrons: one in each of the 3dδ and 3dπ subspaces, e.g., 3dδ_g_^2^ 3dδ_u_^∗2^ 3dδ_g_^2^ 3dδ_u_^∗(↑)^ 3dπ_u_^2^ 3dπ_g_^∗2^ 3dπ_u_^2^ 3dπ_g_^∗(↑)^ 4sσ_g_^2^.

**TABLE 2:** Equilibrium bond lengths, *R*_e_ (Å), binding energies, *D*_e_ (eV), harmonic frequencies, ω_e_ (cm^−1^), and adiabatic transition energies, *T*_e_ (cm^−1^), of electronic states of Ni_2_ calculated at the GVVPT2 level of theory using the cc-pVTZ basis set and reference macroconfigurations κ_3_(**n**) to κ_8_(**n**).

Molecular term	*R*_*e*_ (Å)	*D*_*e*_ (eV)	ω_e_ (cm^−1^)	*T*_*e*_ (cm^−1^)
δπ States computed with κ_4_(**n**)				
1^1^Φ_g_	2.29	1.73	263.7	427.86
1^1^Π_g_	2.29	1.73	269.7	485.09
1^3^Π_g_	2.29	1.72	261.9	518.14
1^3^Φ_g_	2.29	1.72	261.3	546.76
ππ States computed with κ_3_(**n**)				
1^1^Δ_g_	2.23	1.63	242.5	1241.68
2 ^1^Σ_g_^+^	2.28	1.55	240.5	1925.85
States computed with κ_5_(**n**)				
1^3^Δ_u_	3.96	0.02	26.2	33555.78
2 ^3^Σ_u_^+^	3.96	0.02	26.2	33555.91
2 ^1^Δ_g_	3.93	0.03	26.6	34531.63
1 ^3^Σ_g_^+^	3.95	0.03	26.2	39160.31
2 ^3^Σ_g_^+^	3.96	0.03	26.0	39162.41
States computed with κ_6_(**n**)				
2 ^1^Γ_g_	3.73	0.04	26.9	35412.38
3 ^1^Σ_g_^+^	3.73	0.04	26.9	35412.41
States computed with κ_7_(**n**)				
1^5^Φ_u_	3.83	0.03	26.7	33144.35
1^5^Π_u_	3.84	0.03	26.9	33147.67
States computed with κ_8_(**n**)				
1^5^Δ_g_	2.22		249.1	5123.66
2^5^Δ_g_	2.54		150.1	9018.57

At the equilibrium geometries, the EBOs were 0.930 for the singlet 1^1^Φ_g_ and 1^1^Π_g_ states and 0.933 for the 1^3^Π_g_ and 1^3^Φ_g_ states. GVVPT2 predicted the four δπ–hole states to lie energetically in the order 1^1^Φ_g_ < 1^1^Π_g_ < 1^3^Π_g_ < 1^3^Φ_g_, in agreement with the Ref. (Cheskidov et al., 2012) study at the scalar relativistic ACPF, AQCC, MRCI, and MRCI + Q levels of theory. However, our calculations found all three states considered in Ref. (Cheskidov et al., 2012) (i.e., 1^1^Π_g_, 1^3^Π_g_, and 1^3^Φ_g_) to lie some 500 cm^−1^ closer to the ground state; e.g., at the scalar relativistic MRCI + Q level, the 1^3^Φ_g_ state was reported (Cheskidov et al., 2012) as lying 1238 cm^−1^ above the ground state at 2.5 Å while non-relativistic GVVPT2 calculations predicted this state to lie 546.76 cm^−1^ above the ground state at equilibrium. Based on our observation that including scalar relativistic effects increased the energy gap between the 1^1^Σ_g_^+^ and X ^1^Γ_g_ states, it is likely that including such effects in GVVPT2 calculations on the δπ–hole states might lead to increases in corresponding adiabatic transition energies. It is not anticipated, however, that such effects would lead to any change in the energy ordering of the states.

Although lying higher in energy than the δπ–hole states, the ππ–hole states were found to have slightly shorter bond lengths and larger bond strengths than the δπ–hole states. The 1 ^1^Δ_g_ state was 0.06 Å shorter while the 2 ^1^Σ_g_^+^ state was 0.01 Å shorter in bond length than the δπ–hole states. At 2.24 Å, the EBOs of 1 ^1^Δ_g_ and 2 ^1^Σ_g_^+^ were 1.108 and 1.084 respectively (computed with a dozen CSFs); these were slightly higher than the EBOs of all computed δδ–hole and δπ–hole Ni_2_ states. Near equilibrium, the major configurations of these ππ–hole states involved a doubly occupied 4sσ_g_ bonding orbital and a configuration of the 3dπ subspace that had the two π–holes in the same π–orbital; e.g., 3dπ_u_^2^ 3dπ_g_^∗2^ 3dπ_u_^2^ 3dπ_g_^∗0^ 4sσ_g_^2^.

### States of the ^3^F_4_ + ^3^F_4_ and ^3^F_4_ + ^3^D_3_ Manifolds

**FIGURE 6:**
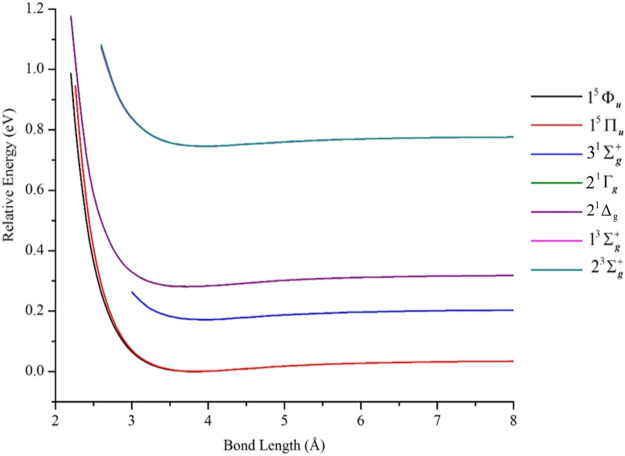
PECs of electronic states of Ni_2_ from the ^3^F_4_ (3d^8^4s^2^) + ^3^F_4_ (3d^8^4s^2^) manifold, computed at the GVVPT2 level of theory using the cc-pVTZ basis set and reference macroconfigurations κ_5_(**n**) to κ_7_(**n**). All energies are plotted relative to the lowest energy value of the 1^5^Φ_u_ term.

Figure 6 contains PECs of states belonging to the ^3^F_4_ + ^3^F_4_ manifold. The data describing these curves are in Table 2. Irrespective of how the model space was partitioned into macroconfigurations [i.e., κ_i_(**n**)], all such states were found to be van der Waals-like with interaction energies ≤0.04 eV. For example, near the equilibrium geometry (i.e., 3.77 Å), the 2 ^1^Γ_g_ and 3 ^1^Σ_g_^+^ states had EBO of only 0.005, while the 1^5^Φ_u_ and 1^5^Π_u_ states had EBOs of 0.003 and 0.00, respectively, at 3.84 Å. These latter quintet states were computed using reference κ_7_(**n**) and found to have the lowest total energies among the computed states of the ^3^F_4_ + ^3^F_4_ manifold; the 1^5^Π_u_ state is 3.312 cm^−1^ less stable than the 1^5^Φ_u_ state at equilibrium. The rest of the PECs in Figure 6 were plotted relative to the 1^5^Φ_u_ PEC.

Lastly, the 1^5^Δ_g_ and 2 ^5^Δ_g_ states of the ^3^F_4_ + ^3^D_3_ manifold were investigated, at short bond lengths only, using reference macroconfiguration κ_8_(**n**). The data for these states are included in Table 2. Whereas the ^3^F_4_ (3d^8^4s^2^) + ^3^F_4_ (3d^8^4s^2^) states are van der Waals-like, the short bond length (2.22 Å) and high frequency (249.1 cm^−1^) of the 1^5^Δ_g_ state is suggestive of significant bonding interaction. At 2.22 Å, the major configuration for this state was 3d_x2-y2_δ_g_^2^ 3d_x2-y2_δ_u_^∗2^ 3d_xy_δ_g_^2^ 3d_xy_δ_u_^∗1^ 3dπ_u_^2^ 3dπ_g_^∗1^ 3dπ_u_^2^ 3dπ_g_^∗1^ 4sσ_g_^2^ 4sσ_u_^∗1^, contributing 50% by weight to the total wave function. At this geometry, the EBO was found to be 1.186. However, one should approach these results for ^3^F_4_ + ^3^D_3_ derived states with some caution, as the macroconfiguration used cannot describe their dissociation limits.

## Conclusion

The GVVPT2 method was used to study low-lying electronic states of Ni_2_. The results indicate, in general, that bonding in these states involves predominantly the doubly occupied 4sσ_g_ bonding orbital with the 3d–3d electrons pairwise spin coupled (e.g., consistent with antiferromagnetism). This statement is corroborated by EBOs that were found to be approximately 1.0 for most states studied and, moreover, states that did not allow this type of interaction [e.g., belonging to the ^3^F_4_ (3d^8^4s^2^) + ^3^F_4_ (3d^8^4s^2^) manifold] were found to be bound only by weak polarization forces with bond orders close to zero. For computed states of the ^3^D_3_ (3d^9^4s^1^) + ^3^D_3_ (3d^9^4s^1^) dissociation limit, all major configurations involved a doubly occupied 4sσ_g_ bonding orbital and a vacant 4sσ_u_^∗^ antibonding orbital. The energy ordering of the computed states of the ^3^D_3_ (3d^9^4s^1^) + ^3^D_3_ (3d^9^4s^1^) manifold is in agreement with previous studies that found the δδ–hole states to lie lowest in energy followed by the δπ–hole and then the ππ–hole states (Rasanen et al., 1987). For the investigated δδ–hole states, the singlets were more stable than the triplet states at the GVVPT2 level of theory. As expected, based on previous GVVPT2 studies of transition metal dimers, all computed PECs are smooth and without unphysical artifacts (e.g., wiggles).

The ground state of Ni_2_ was predicted as X ^1^Γ_g_ in agreement with previous results from other high level *ab initio* methods (Cheskidov et al., 2012; Pou-Amérigo et al., 1994). The determined equilibrium spectroscopic constants of the X ^1^Γ_g_ state, using a cc-pVTZ (or cc-pVTZ-DK) basis, were within the uncertainties of experimental results. The lowest ^1^Σ_g_^+^ state was found to be only 0.002 eV (16.40 cm^−1^) higher than the ground state at the equilibrium geometry. Core-valence correlation was found to be important in the description of low-lying states of Ni_2_ where the inclusion of 3s3p electron correlation at the GVVPT2 level was shown to improve harmonic frequencies and bond energies (e.g., by an increase of 7.5 cm^−1^ in frequency and 0.09 eV in bond energy when 3s3p electron correlation was included in GVVPT2 calculations of the X ^1^Γ_g_ state). Scalar relativistic effects were also shown to be important, especially for dissociation energy, where spectroscopic constants from relativistic calculations were predicted to agree better with reference data (e.g., a decrease of 0.06 Å in bond length, increase of 0.20 eV in bond energy, and an increase of 20.0 cm^−1^ in harmonic frequency when including scalar relativistic effects in calculations of the X ^1^Γ_g_ state). In our previous study (Tamukong et al., 2014) on electronic states of Y_2_, Mn_2_, and Tc_2_, we did not find scalar relativistic effects to be as important for the Mn_2_ molecule as they have proven to be in the present study. The inclusion of spin-orbit coupling effects was previously found (Cheskidov et al., 2012; Rasanen et al., 1987; Pou-Amérigo et al., 1994) to mix the low-lying states of Ni_2_, leading to a 0_g_^+^[^1^Σ_g_^+^(δδ) + ^3^Σ_g_^-^(δδ)] ground state. Since the two curves are close and nearly parallel for all internuclear separations in our study, we expect that including such effects after our scalar relativistic GVVPT2 treatment should lead to a similar mixing of states (i.e., and without change in spectroscopic constants).

The states investigated within the ^3^F_4_ (3d^8^4s^2^) + ^3^D_3_ (3d^9^4s^1^) manifold suggested significant bonding interaction, giving large harmonic frequencies and short bond lengths in comparison with states correlating with the ^3^F_4_ (3d^8^4s^2^) + ^3^F_4_ (3d^8^4s^2^) dissociation limit. Further work on Ni_2_ should explore the ^3^F_4_ (3d^8^4s^2^) + ^3^D_3_ (3d^9^4s^1^) manifold more thoroughly, including possible expansion of the active space. It should be noted, however, that in the present study, we did not observe any significant electron excitations from the valence orbitals to 4p-dominated virtual orbitals. Also, while spin-free relativistic effects are noticeable (especially for bond dissociation energies), the parallelity and small energy separations for states in these manifolds are very similar to those in the ^3^D_3_ (3d^9^4s^1^) + ^3^D_3_ (3d^9^4s^1^) dissociation channel and, again, we do not expect that spin-dependent relativistic effects would change PECs appreciably, although current capabilities of our GVVPT2 code did not let us explore this.

In summary, the present study showed that Ni_2_ does not form strong bonds with atomic configurations in which the 4s subshell is fully filled. This observation is consistent with other studies, by us and by others, of transition elements of the first row. Bonding in these systems is generally favored by atomic configurations that involve at least one of the participating atoms in an excited state (3d^*n*+1^4s^1^). For example, in our previous study (Tamukong et al., 2012) of the low-lying electronic states of Sc_2_, Cr_2_, and Mn_2_, the lowest states of Sc_2_ were shown to correlate with the ^2^D (3d^1^4s^2^) + ^4^F (3d^2^4s^1^) dissociation asymptote, while those of Cr_2_ correlated with the ^7^S (3d^5^4s^1^) + ^7^S (3d^5^4s^1^) dissociation limit. However, bonding in transition metal dimers is subtle and the most stable states of Mn_2_ were obtained from weakly coupled ^5^D (3d^5^4s^2^) + ^5^D (3d^5^4s^2^) ground state Mn atoms, similar to the ^3^F_4_ (3d^8^4s^2^) + ^3^F_4_ (3d^8^4s^2^) coupling of ground state Ni atoms. Our results provide further evidence that GVVPT2, with sf-X2C treatment of relativistic effects, predict electronic excitation and bond energy trends in the first row transition metals consistent with experiment and the highest level *ab initio* calculations. In the present case, the quasidegeneracy of the ^3^F and ^3^D states of the Ni atom demonstrates that GVVPT2 can successfully be used for a system with an extraordinarily dense manifold of states, which generally requires computationally intensive variational treatments.

## Data Availability

The raw data supporting the conclusions of this article will be made available by the authors, without undue reservation.
